# The Multiple Dimensions of Participation: Key Determinants of Nutrition Intervention Outcomes

**DOI:** 10.1016/j.cdnut.2022.100001

**Published:** 2022-12-17

**Authors:** Grace S. Marquis, Stephanie L. Martin, Anna Lartey

**Affiliations:** 1 School of Human Nutrition, McGill University, Montreal, Québec, Canada; 2 Department of Nutrition, Gillings School of Global Public Health, University of North Carolina at Chapel Hill, Chapel Hill, NC, USA; 3 Department of Nutrition and Food Science, University of Ghana, Legon, Ghana

**Keywords:** participation, community-based intervention, stakeholder, engagement, evaluation

## Abstract

Nutrition research benefits from broad and intensive participation by stakeholders. The articles in this series demonstrate that understanding participation is complex because it incorporates the dimensions of stakeholders, activity, time, and intensity. Early involvement in research can help prioritize the problems to be addressed, refine the specific research question, and determine acceptable community-based approaches to be used in an intervention. The included studies examined the construct of participation and the diverse means by which it can be measured. They demonstrated how knowledge gained from early participation influenced the direction of interventions and increased relevancy for the community. The researchers assessed participation intensity during the intervention phase to help explain project outcomes and provide estimates of the magnitude of the effect that could be achieved if high-level participation of stakeholders was universal. In addition, participation in the analysis process was a key component of some of the articles in this series, demonstrating the richness of understanding that can be obtained through collaborative analyses. The included papers provide insight into how to define and measure participation, how to explore approaches to encourage participation of direct and indirect beneficiaries, and how participation at different time points and by different stakeholders can validate and support interventions and enhance effectiveness. As such, the series serves as a valuable reference to researchers, program and policy designers, implementers, and evaluators to increase the benefits of community-based interventions for nutrition outcomes.

This series examines the construct of participation, how it is defined, measured, and integrated into all stages of nutrition research projects and programs and how it influences intervention outcomes. The articles throughout this series consider the wisdom gained by opening the research process to a participatory collaboration and the limitations of our interventions if we do not facilitate, document, and integrate it into the analysis. There are complex interactions among environments in which vulnerable populations live, their economic and social conditions, and individual characteristics that determine their nutritional status [1]. While estimating the benefits of an intervention, researchers often assume that the delivery of the activities and participation by recipients occurred as planned. However, many of the factors that have been reported to be associated with nutrition outcomes—e.g., maternal education [2], social support for caregivers [3], and household wealth [4]—may influence whether an individual enrolls in an intervention in the first place and their level of participation throughout a project, making the relationships even more complex. For example, Baik et al. [5] reported in this series that participation in the Positive Deviance/Hearth-Interactive Voice Calling program, in part, was facilitated by social support, whereas it was hindered by low financial resources and women’s education in Cambodia. Abdu et al. [6] noted the association between women's empowerment and women’s membership in farmer-based organizations, which are important focal points for nutrition-sensitive interventions in rural regions of Ghana. Similarly, Manandhar et al. [7] identified maternal education and self-efficacy as well as household characteristics, such as sex of the household head, to be associated with active participation in volunteer-led, health groups of mothers in communities in Nepal.

Process evaluations help assess the extent and characteristics of participation by recipients in addition to the fidelity of planned activities [8]. A process evaluation can inform when participation occurred (from identification of the research question to dissemination of results), the intensity of engagement, and the proximity of the participating individual to the intervention (i.e., intended vs. indirect beneficiary). Robert and Feijoo [9] provided a narrative review of the participation continuum in community-based nutrition interventions and suggested a framework to stimulate future research.

Community participation at the early stages of an intervention is recommended [10] and typically involves community engagement as an exercise to inform residents about upcoming activities, receive permission from gatekeepers to enter an area, and promote recruitment. However, this initial engagement is less commonly used to identify the problem to be addressed and decide on the intervention approach and activities to develop. This series provides examples of different approaches for early involvement of participants to define the problem, refine the research question, and determine how it could be addressed. For example, Colecraft et al. [11] described a qualitative inquiry in which a wide range of stakeholders (from the national government staff to rural women farmers) worked together with researchers to develop a conceptual framework for barriers to the inclusion of animal source foods in children’s diets. This framework then guided proposal writing for a nutrition-sensitive agriculture intervention. Klemm et al. [12] reported a participatory process of supporting Tanzanian regional and district nutrition officers to develop action teams and test multisectoral nutrition approaches that reflect the local context. A feasibility assessment of urban agriculture in Kenya included beneficiaries with diverse levels of engagement in the program to examine the facilitators of and barriers to full project participation [13]. The assessment was planned to contribute to the design of scale-up of sack agriculture for improving food security and diet diversification. As part of a larger project on nutrition counseling tools in Ghana, Sandow et al. [14] conducted a qualitative study in caregivers of young children as well as facility- and community-based health workers. The caregivers confirmed the importance of health provider counseling for their knowledge and practices, and health workers provided recommendations on staff training and the integration of responsive feeding education into future training materials. McEachern et al. [15] reported a community-based participatory case study in which local organizations came together with indigenous community members to discuss community priorities regarding diet, barriers that they faced, and planned activities that could be performed to address their concerns. van Zadelhoff and Haisma [16] noted the value of including community perspectives on growth monitoring in their evaluation of growth monitoring manuals from India, the Netherlands, and Tanzania, recognizing the need to have engaged relevant stakeholders throughout the research process. Finally, Gonzales Martinez et al. [17] reported 2 nutrition-sensitive interventions (water, sanitation, and hygiene as well as health infrastructure) in Vietnam. There was a positive association between child growth (using a multiple dimension index) and community engagement in the design stage for the water, sanitation, and hygiene intervention. The knowledge gained from early participation in these studies could influence the direction of later interventions and programs, thus helping to increase their relevancy for the community.

An assessment of participation intensity may help explain project results and provide an estimate of the magnitude of the effect that could be achieved with high levels of engagement. In a quasiexperimental study by Scott et al. [18], the difference in maternal and child nutrition outcomes between villages with and without an intensive nutrition behavior change communication intervention added to standard activities of self-help groups in India was examined. The limited benefits (improved knowledge about and consumption of animal foods) associated with the intervention were attributed partly to substantial participation barriers, which were documented in low attendance at self-help group meetings. Flax et al. [19] reported Nigerian parents’ exposure to a social and behavior change communication intervention about complementary feeding. Although the exposure was low overall, their children’s intake of fish and eggs as well as other diet quality indicators were positively associated with maternal, but not paternal, participation indicators (e.g., participation in a home visit and attending a meeting). An article by Dallmann et al. [20], in which secondary data from a randomized controlled trial in Ghana were analyzed, demonstrated a difference in the effect of an integrated agriculture (poultry production and gardens) and nutrition education intervention on children’ egg consumption and linear growth, depending on the intensity of women’s participation. The estimate of the length- or height-for-age z-score among women with a high level of participation was double that of the intention-to-treat estimate obtained from the whole sample. In this study, the participation level was a composite index of 5 indicators obtained from regularly documented data (e.g., meeting attendance) in addition to an evaluation by the project staff (e.g., women’s contribution to the discussion during meetings). Programs use different tools to develop indicators of recipients’ participation. Given the challenges associated with assessment, the documentation of participation is likely to diminish as it moves farther away from that which is easily countable. The attendance in group meetings may be easily recorded, whereas individual compliance with project-recommended animal feeding and care strategies, for example, may be quite challenging to monitor and report. Creative approaches that are integrated into existing systems may help. For example, Ojwang et al. [21] trained teachers to document their students’ dietary intakes daily using diet diversity registers, providing an indicator that reflected participation intensity and compliance with the intervention’s promotion of the consumption of orange-fleshed sweet potatoes.

Participation in the analysis process was a key component of some of the articles in this series. Boedecker et al. [22], in their study, used qualitative methods to understand why and how an agricultural intervention improved maternal and child nutrition in Kenya. Community participants were a part of focus group discussions to examine the pathways of changes that they experienced over time, from initial planning workshops to the implementation of agricultural activities. This knowledge was complemented through interviews with the perspectives of involved stakeholders from local institutions. Furthermore, Daniel et al. [23] used qualitative methods to understand the perceptions and experiences of caregivers of children treated for severe acute malnutrition who participated in a nutrition, water and sanitation, and psychosocial stimulation counseling program in Malawi. The study was important for understanding which components of the program the participants valued, even though it was ineffective in improving nutritional and developmental outcomes. The discussions provided important lessons to be incorporated into future interventions. Two articles used a participatory video as a means of engagement of the youth in the analysis and dissemination stages. In a study by Rao et al. [24], the Indian youth used film making to explore their concerns about locally available foods to improve diet diversity. Over time, the filmmaker youth developed confidence in their abilities to document local issues and their in-person and YouTube channel audiences became engaged in the discussion of the relevance of the films, generating new themes for future work. After disseminating their films to their peers and communities, Ghadirian et al. [25] used the Most Significant Change methodology to further engage Ghanaian female adolescent participants and local stakeholders to reflect and prioritize the key outcomes of the film-making project. This exercise helped to demonstrate the differences in the outcome priorities of female adolescents and those of adult local stakeholders that are important to note for future interventions.

The articles in this series provide insight into how to define and measure participation, how to explore approaches to encourage participation of direct and indirect beneficiaries, and how participation at different time points and by different stakeholders can validate and support interventions and enhance effectiveness. These researchers recognized that participation is complex. It is not related solely to direct beneficiaries; there are many stakeholders with unique patterns of participation that may influence the process of research. These include the influence of the staff on the fidelity of intervention activities, initiation and level of commitment to the activities by project recipients, and social actors that reinforce and compete for that commitment. Researchers need to understand the complexity of the system in which an intervention occurs and be thoughtful of the pathways by which these different types of participation may influence the nutrition outcomes. Goh et al. [26] examined the possibility of unexpected consequences, such as deterioration of the quality of childcare with an increase in time demand, among women participating in a nutrition-sensitive agriculture intervention in Ghana. They demonstrated that project participation did not adversely affect the duration or quality of care of young children despite the increased time needed to complete project activities. Walker et al. [27] suggested that nutrition researchers use methods from systems science while examining barriers to the adoption and sustainability of complex nutrition interventions. With documentation, these outcomes can be better understood.

In summary, we need to start with a systems approach to appreciate the relationships, complexity, and dynamic environment in which interventions occur. We need effective and culturally acceptable interventions that can reasonably be expected to affect outcomes. Furthermore, we need to identify innovative approaches that will increase and promote high-quality participation of different actors throughout the intervention. We need to document participation using different lenses and methodologies that will capture its diversity and influential pathways. Finally, we need to use mixed methods and multiple analysis approaches to understand how participation influences outcomes of interest and possible unintended consequences. With this knowledge, we will have a better estimate of the potential impact that nutrition interventions can achieve. This series serves as a valuable reference to researchers, program and policy designers, implementers, and evaluators to better understand and improve various dimensions of participation and ultimately increase the impact of the program on nutrition outcomes.

## Author disclosures

GSM is an Associate Editor for *Advances in Nutrition* and *Current Developments in Nutrition* series’ Deputy Editor and played no role in the journal’s evaluation of the manuscript. SLM and AL are the series’ Guest Editors for *Current Developments in Nutrition* and played no role in the journal’s evaluation of the manuscript.
